# Rat *Mammary carcinoma susceptibility 3* (*Mcs3*) pleiotropy, socioenvironmental interaction, and comparative genomics with orthologous human *15q25.1-25.2*

**DOI:** 10.1093/g3journal/jkac288

**Published:** 2022-10-31

**Authors:** Emily L Duderstadt, David J Samuelson

**Affiliations:** Department of Biochemistry & Molecular Genetics, University of Louisville School of Medicine, Louisville, KY 40202, USA; Department of Biochemistry & Molecular Genetics, University of Louisville School of Medicine, Louisville, KY 40202, USA; James Graham Brown Cancer Center, University of Louisville School of Medicine, Louisville, KY 40202, USA

**Keywords:** rat *Mcs3*, breast cancer susceptibility, genotype by environment interaction, mammary gland development, body mass, human *15q25.1-25.2*

## Abstract

Genome-wide association studies of breast cancer susceptibility have revealed risk-associated genetic variants and nominated candidate genes; however, the identification of causal variants and genes is often undetermined by genome-wide association studies. Comparative genomics, utilizing *Rattus norvegicus* strains differing in susceptibility to mammary tumor development, is a complimentary approach to identify breast cancer susceptibility genes. *Mammary carcinoma susceptibility 3* (*Mcs3*) is a Copenhagen (COP/NHsd) allele that confers resistance to mammary carcinomas when introgressed into a mammary carcinoma susceptible Wistar Furth (WF/NHsd) genome. Here, *Mcs3* was positionally mapped to a 7.2-Mb region of *RNO1* spanning *rs8149408* to *rs107402736* (*chr1*:143700228–150929594, build 6.0/rn6) using WF.COP congenic strains and 7,12-dimethylbenz(a)anthracene-induced mammary carcinogenesis. Male and female WF.COP-*Mcs3* rats had significantly lower body mass compared to the Wistar Furth strain. The effect on female body mass was observed only when females were raised in the absence of males indicating a socioenvironmental interaction. Furthermore, female WF.COP-*Mcs3* rats, raised in the absence of males, did not develop enhanced lobuloalveolar morphologies compared to those observed in the Wistar Furth strain. Human *15q25.1-25.2* was determined to be orthologous to rat *Mcs3* (*chr15*:80005820–82285404 and *chr15*:83134545–84130720, build GRCh38/hg38). A public database search of *15q25.1-25.2* revealed genome-wide significant and nominally significant associations for body mass traits and breast cancer risk. These results support the existence of a breast cancer risk-associated allele at human *15q25.1-25.2* and warrant ultrafine mapping of rat *Mcs3* and human *15q25.1-25.2* to discover novel causal genes and variants.

## Introduction

Breast cancer is the most frequently diagnosed cancer among women and a leading cause of premature mortality (DeSantis *et al.* 2014; Oeffinger *et al.* 2015). Susceptibility to this complex disease is modulated by genetic, epigenetic, and environmental factors. The genetic component of breast cancer susceptibility is controlled by an array of high, moderate, and low penetrance risk-associated mutations and variants (Pharoah *et al.* 2002). Breast cancer susceptibility is a polygenic model with many genes contributing. High to moderate penetrant mutations in breast cancer predisposition genes, such as *BRCA1* and *BRCA2*, are rare in large populations and account for only 5–10% of total breast cancer cases (Carroll *et al.* 2008; Economopoulou *et al.* 2015). A majority of genetic risk is accounted for by additive effects of low-penetrant variants in noncoding DNA regulatory elements (Breast Cancer Association Consortium 2006; Mavaddat *et al.* 2010). Causal genes at most susceptibility loci remain unknown (Breast Cancer Association Consortium 2006; Smith *et al.* 2006; Ghoussaini and Pharoah 2009; Mavaddat *et al.* 2010; Michailidou *et al.* 2017). In addition to difficulty in determining causal genes, many nominally associated variants are identified in genome-wide association studies (GWAS) that do not meet stringent *P*-values required for genome-wide significance. These associations mark potential true positive associations. One way to evaluate these nominally associated alleles and identify causal genes is with comparative genomics approaches that incorporate experimental organisms that models breast cancer susceptibility (Gould 2009; Colletti *et al.* 2014; Sanders and Samuelson 2014).

The laboratory rat (*Rattus norvegicus*) presents one of the best in vivo human breast cancer models, as induced rat mammary and female breast carcinomas have similar histopathological stages and features, including epithelial ductal cell origin, progression, and hormone responsiveness and nonresponsiveness (Russo *et al.* 1990; Nandi *et al.* 1995; Russo and Russo 1996; Shepel *et al.* 1998; Samuelson *et al.* 2007; Gould 2009; Sharma *et al.* 2011). Inbred rat strains differ in their susceptibility to 7,12-dimethylbenz(a)anthracene (DMBA)-, *N*-methyl-*N*-nitrosourea-, and estrogen-induced mammary carcinogenesis. Many rat strains have been used to discover quantitative trait loci (QTLs) that control mammary cancer susceptibility. These loci are named *Mammary carcinoma susceptibility* (*Mcs*) and *Estrogen-induced mammary cancer* (*Emca*) QTLs (Hsu *et al.* 1994; Shepel *et al.* 1998; Lan *et al.* 2001; Gould *et al.* 2004; Quan *et al.* 2006; Schaffer *et al.* 2006; Ren *et al.* 2013). Genetic linkage analyses of crosses between DMBA-induced mammary carcinoma susceptible Wistar Furth (WF) and resistant Copenhagen (COP) strains resulted in 4 predicted *Mcs* QTLs termed *Mcs1*, *Mcs2*, *Mcs3*, and *Mcs4* (Hsu *et al.* 1994; Shepel *et al.* 1998). The *Mcs1*, *Mcs2*, and *Mcs3* QTLs were physically confirmed using congenic rat strains with the COP resistance-associated allele of interest introgressed into a susceptible WF genome (Haag *et al.* 2003; Sanders *et al.* 2011; Le *et al.* 2017). Rat *Mcs3* has been physically confirmed as an independently acting QTL and delimited to a 29.4-Mb genomic region of rat chromosome *one* (*RNO1*) (Le *et al.* 2017). In this article, we report WF.COP congenic strain studies that further delimit the *Mcs3* resistance allele to a 7.2-Mb region of *RNO1*. We also report that *Mcs3* females have reduced body mass and different mammary gland morphology compared to WF females. Interestingly, these latter phenotypes only manifest when females are housed in the absence of males. In our comparative genomics analysis of *Mcs3* and orthologous human *15q25.1-25.2*, we found previously reported breast cancer and body mass-associated variants.

## Methods

*Congenic rat strains* were established and maintained in an Association for the Assessment and Accreditation of Laboratory Animal Care-approved facility on a 12-h light/dark cycle and provided LabDiet 5001 Rodent Diet (PMI Nutrition International) and water ad libitum. The University of Louisville Animal Care and Use Committee approved all animal protocols used in this study. Rat *Mcs3* WF.COP congenic strains were developed with a WF/NHsd genome and COP/NHsd alleles introgressed at selected loci spanning the previously published *Mcs3* QTL (Le *et al.* 2017). Rat WF.COP-*Mcs3* resistance-associated strain D had a COP allele spanning *RN01* from single-nucleotide variant (SNV) *rs8149408* to SNV *rs105131702* (Le *et al.* 2017). Heterozygous (COP/WF) WF.COP-*Mcs3*^D^ males at congenic generation N16 were backcrossed to inbred WF/NHsd females (Envigo). Informative N17 recombinants were backcrossed to WF/NHsd animals for expansion, and subsequent N18 offspring were inbred using brother–sister matings to establish unique WF.COP-*RNO1* congenic strains for this study. Sequence information and genomic locations of genetic markers defining COP alleles in these strains are available at the UCSC Genome Browser (www.genome.ucsc.edu), the Rat Genome Database (http://rgd.mcw.edu/), and Supplementary Tables 1 and 2. A WF.COP strain that did not inherit COP alleles at the N18 generation was maintained in house to serve as a WF/NHsd mammary carcinoma susceptible strain.

*Genotyping* was completed using standard PCR-based genotyping methods previously described (Samuelson *et al.* 2003). Briefly, microsatellite marker primers were used to PCR amplify genomic DNA at the congenic allelic segment ends and intervals within. Fast-PCRs underwent denaturation of 95°C for 10 s, followed by 40 cycles of 94°C for 0 s and 63°C for 8 s, and an extension at 72°C for 30 s on an Applied Biosystems Veriti Fast Thermal Cycler. Amplified DNA was resolved on 3% high-resolution agarose gels, stained with ethidium bromide or SybrGold, scanned with a digital imager, and visualized with ImageQuant (Amersham Biosciences). Segments containing 4 WF/COP informative SNVs were PCR-amplified and sequenced using dideoxy sequencing by the University of Louisville, Center for Genetics and Molecular Medicine DNA sequencing core with an ABI PRISM 3130XL Sequence Detection System (Life Technologies).

*Phenotypes* of female WF.COP congenic rats, along with female WF/NHsd rats (Envigo), included as mammary cancer-susceptible controls, were determined using females that were moved at weaning to a standard housing room that did not contain males unless otherwise noted. Mammary carcinogenesis was induced by administering 65 mg DMBA per kg body mass (ACROS Organics; Fisher Scientific, Pittsburgh, PA, USA) suspended in sesame oil as a single oral gavage at 50–55 days of age. Mammary carcinomas ≥3 × 3 mm^2^ were counted at 15 weeks post-DMBA administration. Sample number (*n*) and congenic generation in parentheses of females used for each strain were *n* = 28 WF.COP-WF/NHsdUl (N18-F2, -F3, -F4), *n* = 20 strain H (N18-F2, -F3, -F4), *n* = 26 strain I (N18-F2, -F3, -F4), *n* = 30 strain J (N18-F2, -F3, -F4), and *n* = 25 strain K (N18-F2, -F3, -F4).

To measure body mass, congenic WF.COP-*Mcs3* strain J (*Mcs3^J^*) and WF/NHsd male and female rats in regular housing with both males and females present were weighed at 4, 8, and 12 weeks of age (*n* = 31 WF males, *n* = 34 WF females, *n* = 30 *Mcs3^J^* males, and *n* = 29 *Mcs3^J^* females) using a standard digital scale. In a follow-up experiment, WF.COP-*Mcs3^J^* (*n* = 24) and WF/NHsd (*n* = 15) females were housed without males present in the room after weaning and weighed at 8 and 12 weeks of age. The congenic generations of these rats were N18-F3, -F4, and -F5.

*Tissue collection and histological analysis* was completed using 4 representative mammary tumors (invasive ductal carcinomas). Mammary carcinomas were induced with DMBA at 50–55 days and resected 15 weeks post-DMBA from euthanized (CO_2_ asphyxiation) females of each WF.COP congenic and WF/NHsd strain. Nondiseased abdominal-inguinal mammary glands were collected from euthanized WF/NHsd (N18F10) and *Mcs3^J^* females (N18F9) that were housed in an animal room without males and aged 30–35 days (*n* = 3 per strain), 50–55 days (*n* = 7 per strain), and 12 weeks (*n* = 3 per strain). Tissues were fixed overnight in 10% neutral buffered formalin, processed using a standard tissue processor, and paraffin embedded. Paraffin tissue blocks were sectioned at 5 µm and placed on Superfrost Plus microscope slides using a rotary microtome (Thermo HM 355S). Slides were deparaffinized and rehydrated using 3 separate xylene washes and 4 washes in 100% ethanol, 90% ethanol, 80% ethanol, and DI water. Tissues were stained with hematoxylin (H) (Fisher Scientific 469803) for 3 min, rinsed in running distilled (DI) water, dipped in bluing reagent (Fisher Scientific 22-220-106), and rinsed in running DI water. Tissues were stained with Eosin-Y (E) (Fisher Scientific 22-220-104) for 30–45 s. Tissues were dehydrated in washes of 90% ethanol, 100% ethanol, and 3 separate xylene washes before using Permount Mounting Medium (Electron Microscopy Sciences 17986-05) and placing a coverslip.

*Mammary gland lobuloalveolar morphology* was quantified in FFPE, H&E-stained mammary gland cross-sections from 12-week-old WF/NHsd (N18F10) and *Mcs3^J^* (N18F9) females that had been housed in regular housing with either males present or absent after weaning (*n* = 3 rats per group). Tissue morphology of interest was outlined, and % area quantified using open-source ImageJ Fiji software. Averages of 3 representative fields of view per rat were used for analysis.

*Comparative genomics* involved interrogating the rat *Mcs3* and human orthologous sequences. Human orthologous syntenic regions that mapped to the delimited rat *Mcs3* were identified using the convert function of the UCSC Genome Browser. The *R. norvegicus* reference genome build version RGSC 6.0/rn6 and *Homo sapiens* version GRCh38/hg38 were used. Rat *Mcs3* and human orthologous syntenic region annotated genes and noncoding DNA were curated using the table browser function of the UCSC Genome Browser. The NHGRI-EBI Catalog of human genome-wide association studies was used to identify breast cancer disease and risk correlated traits that had *P*-values of 1 × 10^−7^ or less for association. NCBI-PubMed was searched to identify rat *Mcs3* and human orthologous genes with published associations to breast cancer.

*Statistical analysis* of mammary carcinoma multiplicity data was performed using nonparametric Kruskal–Wallis and Dunn’s post hoc tests. Body mass data collected at the time of mammary tumor multiplicity counts were analyzed by ANOVA and Dunnett’s post hoc test. Body mass data collected at different ages were analyzed by 2-way ANOVA with strain and age as independent variables, followed by Tukey’s post hoc test. Mammary gland lobuloalveolar cross-sectional area percentage quantifications were arcsine transformed and analyzed using a 2-tailed unpaired *t*-test. *P*-values ≤0.05 were considered statistically significant. All statistical analyses were done using GraphPad Prism version 9.2.0 for Windows, GraphPad Software, La Jolla, CA, USA. All data are presented within the article and supplementary files.

## Results

### Mapping and pleiotropy of *Mcs3*

**Fig. 1.**
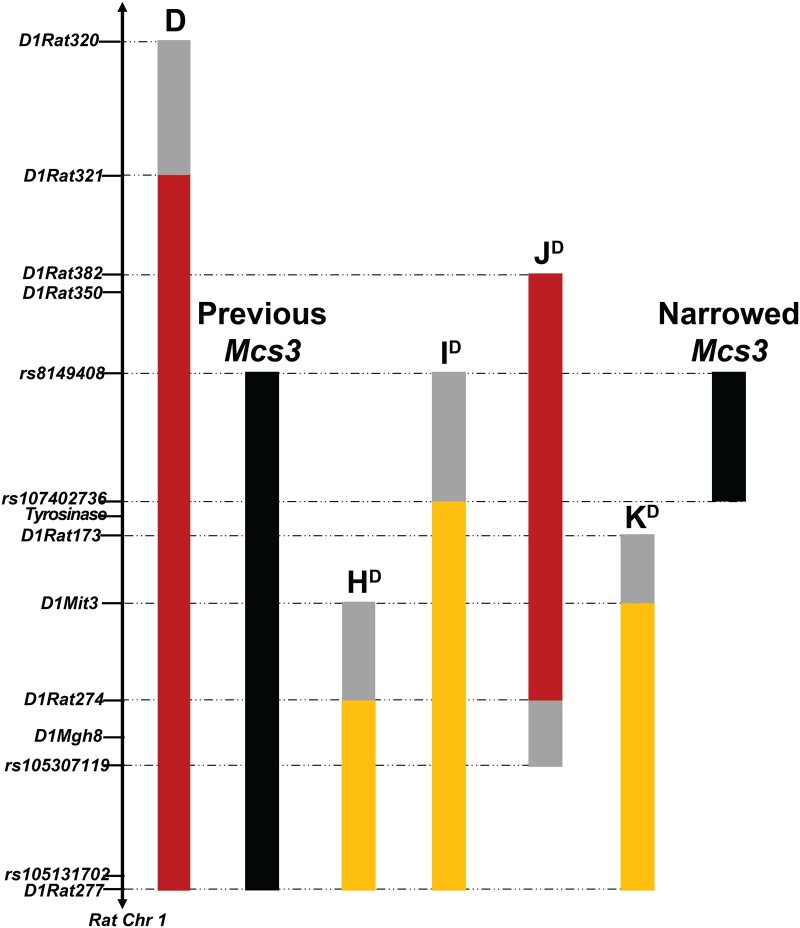
Map of WF.COP congenic alleles delimiting *Mcs3* to 7.2 Mb of *R. norvegicus* chromosome *1* (*RNO1*). Informative WF/COP rat genomic markers are listed on the vertical axis and placed in relative position along *RNO1.* Congenic strains (WF.COP) are represented by the respective COP allele (colored vertical bars), a strain-specific letter, and the WF.COP strain from which it was derived (superscripted letter). WF.COP strains with a mammary carcinoma resistance phenotype are denoted by red bars (strains D and J), while susceptible strains are shown with yellow bars (strains H, I, and K). Gray bars represent genomic regions of unknown genotype. WF.COP strain D was published previously by Le *et al.* as an *Mcs3* containing strain.

**Fig. 2.**
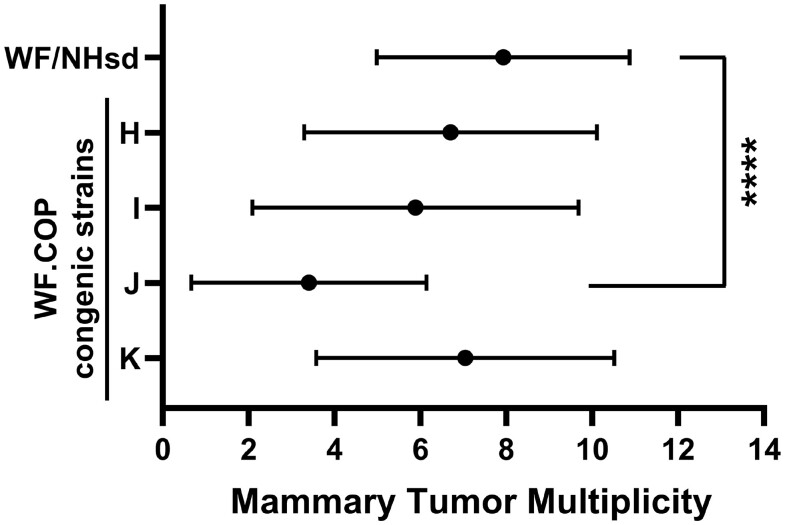
Mammary carcinoma susceptibility phenotypes. Mean ± SD mammary tumor multiplicity for WF.COP and susceptible control WF/NHsd strains. Mammary tumors were counted at 15 weeks post-DMBA administration. Dunn’s nonparametric post hoc tests, comparing each line to the WF/NHsd strain phenotype, were performed following a significant Kruskal–Wallis test (*P* < 0.0001). Strain J (WF.COP-*Mcs3^J^*) was the only strain that was significantly different from the WF/NHsd strain (*P* < 0.0001).

Rat *Mcs3* was physically mapped using 4 WF.COP congenic strains that contained different COP donor segments of *RNO1* (Fig. 1). Together these segments covered a 29.4-Mb region of *RNO1* that previously defined *Mcs3* (Shepel *et al.* 1998; Le *et al.* 2017). Genomic locations and genetic markers of COP alleles contained in each WF.COP congenic strain along with respective mammary tumor multiplicity phenotypes are in Table 1. Mammary carcinoma susceptibility phenotype statistics of these WF.COP and WF/NHsd strains are displayed graphically in Fig. 2 and Supplementary Fig. 1. The congenic recipient strain (WF/NHsd) is known to have a high susceptibility phenotype. These females (*n* = 28) developed an average of 7.9 ± 2.9 (mean ± SD) mammary tumors per rat in this study. This phenotype was similar to previously published phenotypes for this strain (Veillet *et al.* 2011; denDekker *et al.* 2012; Le *et al.* 2017). WF.COP congenic strains H, I, and K developed 6.7 ± 3.4 (*n* = 20), 5.9 ± 3.8 (*n* = 26), and 7.0 ± 3.5 (*n* = 25) mammary tumors per rat, respectively. None of these strains were statistically different in susceptibility from the WF/NHsd strain (*P*-values = 0.766, 0.0932, and >0.999, respectively). WF.COP strain J females (*n* = 30) developed 3.4 ± 2.7 mammary tumors per rat, which was significantly different than WF/NHsd females (*P* < 0.0001). Collectively, this congenic strain panel delimited rat *Mcs3* to a 7.2-Mb region of *RNO1* spanning from genetic markers *rs8149408* to *rs107402736* (*RNO1*:143700228–150929594, rat reference genome build RGSC 6.0/rn6).

**Table 1.** Genomic intervals and mammary carcinoma susceptibility phenotypes of WF.COP congenic strains.

Strain	Markers defining congenic interval	Genomic interval^a^	Interval size (Mb)	Percent overlap with previous *Mcs3*^b^	Mean (SD) mammary carcinomas per rat	*n*	*P*-Value comparison to WF/NHsd
D^b^	D1Rat320/D1Rat65		71	100	2.8 (2.3)	19	–
H	D1Rat274/D1Rat277	chr1:161677683–171716714	10	34	6.7 (3.4)	20	0.7666
I	rs107402736/D1Rat277	chr1:150929594–171716714	20.8	71	5.9 (3.8)	26	0.0932
J	D1Rat382/D1Rat274	chr1:137503201–161677683	24.2	61	3.4 (2.7)	30	<0.0001
K	D1Mit3/D1Rat277	chr1:156446196–171716714	15.3	52	7.0 (3.5)	25	>0.9999
WF/NHsd	–				7.9 (2.9)	28	–

a *R. norvegicus* genome build version RGSC 6.0/rn6.

b Le *et al.* (2017).

**Fig. 3.**
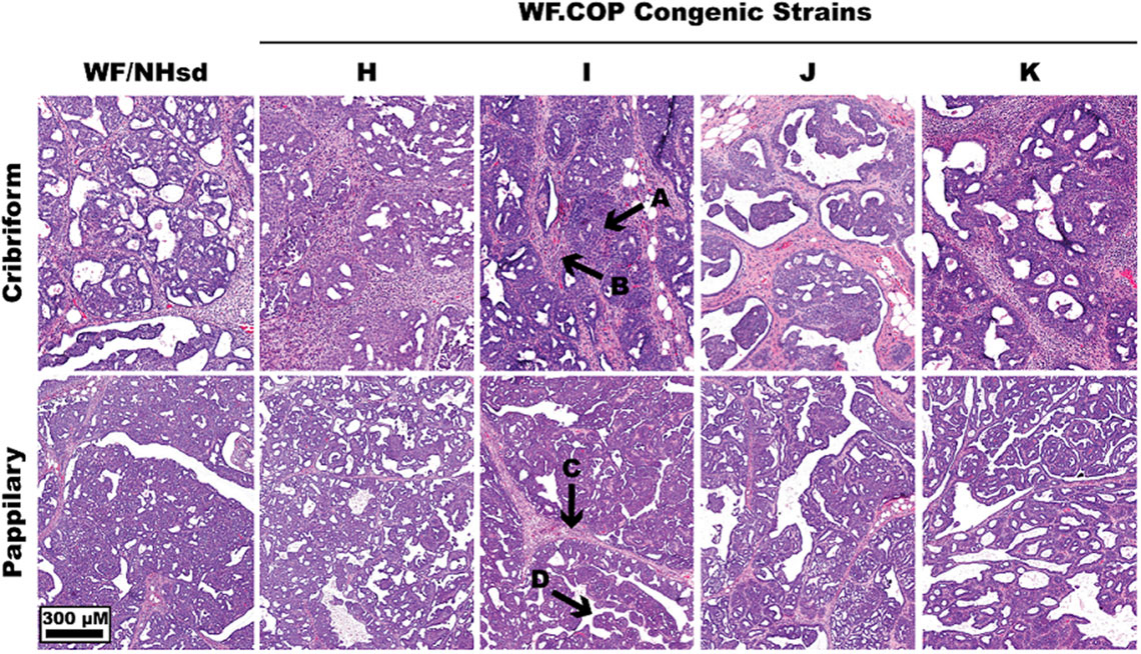
Histology of WF.COP congenic strain mammary tumors. All WF.COP strains tested developed expected DMBA-induced invasive cribriform and papillary carcinomas. Images are H&E-stained DMBA-induced representative mammary tumors forming in WF/NHsd and WF.COP strains. The top row is representative cribriform patterns, defined by neoplastic epithelial cell tumor nests (arrow A) within a surrounding desmoplastic stroma (arrow B). The bottom row contains representative papillary patterns, defined by fibrovascular cores that form a network throughout the tumor (arrow C), with neoplastic epithelial cells growing to expand the lesion via papillary projections (arrow D). Images were taken at 40× magnification on an Aperio ImageScope CS2.

Histopathological analysis of mammary tumors revealed no discernable differences between WF.COP congenic and WF strains. Mammary tumors were invasive ductal carcinomas, with a majority being invasive papillary and invasive cribriform carcinoma subtypes (Fig. 3).

**Fig. 4.**
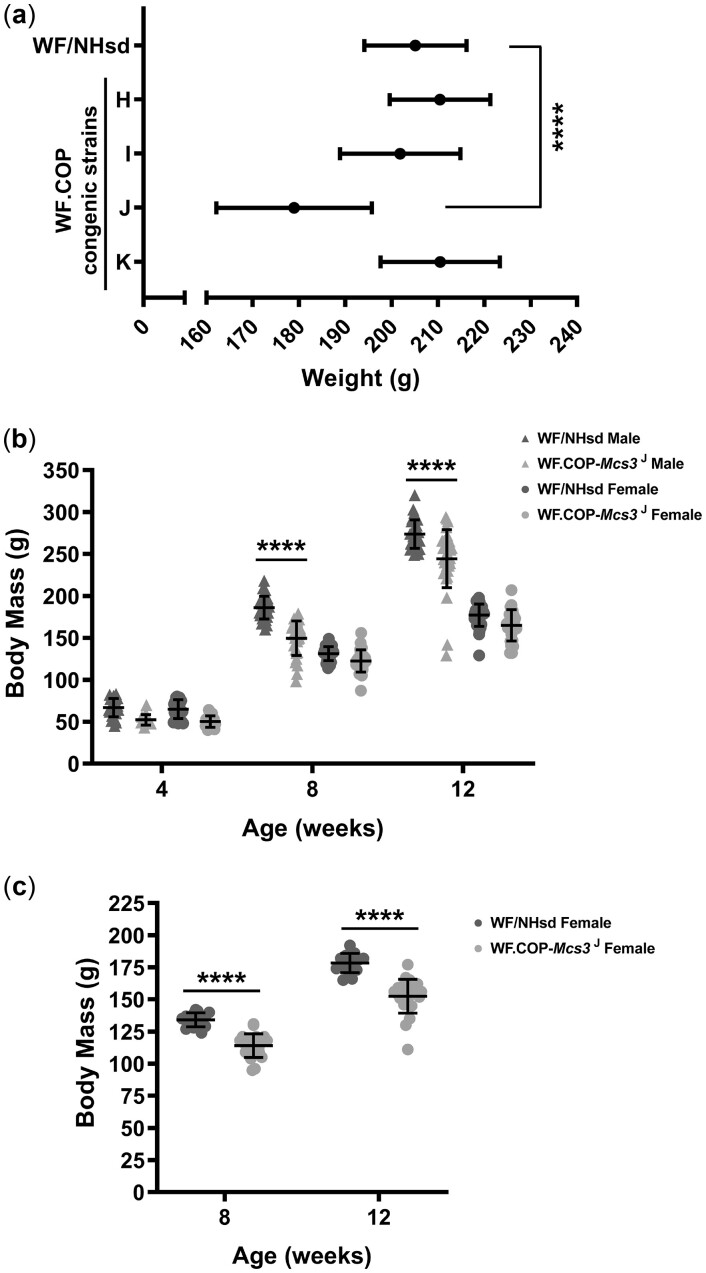
Effects of *Mcs3* and social environment on body mass. a) Mammary carcinoma-resistant WF.COP-*Mcs3^J^* females had lower body mass than susceptible WF/NHsd strain females at 23 weeks of age, which was when mammary carcinoma susceptibility phenotypes were measured (tumor multiplicity). A 1-way ANOVA was performed (*P* < 0.0001), followed by Dunnetts’s post hoc tests comparing each strain to the control WF/NHsd strain. Strain J was significantly different from WF/NHsd (*P* < 0.0001). b) The effect of *Mcs3* on female body mass was lost when females were raised in social environment containing males. Male and female rats from WF/NHsd and WF.COP-*Mcs3^J^* strains were weighed at weaning age (4 weeks), puberty (8 weeks), and breeding age (12 weeks). WF.COP-*Mcs3^J^* males had significantly reduced body mass compared to WF/NHsd males at 8 and 12 weeks of age (2-way ANOVA followed by Tukey’s multiple comparisons post hoc test, *P* < 0.0001), but no differences in body mass were found between females at 4, 8, and 12 weeks of age. c) The effect of *Mcs3* on body mass was evident at 8 and 12 weeks of age when, at weaning, females were placed in a social environment that did not contain males (2-way ANOVA, *P* < 0.0001).

Pleiotropy of the *Mcs3* allele contained in WF.COP strain J (*Mcs3^J^*) was observed in this study. Rat *Mcs3^J^* females, exposed to DMBA, had a body mass of 179 ± 17 g (mean ± SD) at 23 weeks of age, which was significantly lower than age-matched and DMBA-exposed WF/NHsd females that had a body mass phenotype of 201 ± 21 g (*P* < 0.0001) (Fig. 4a). Mammary cancer-susceptible WF.COP congenic strains K, H, and I had body mass phenotypes of 210 ± 13, 210 ± 11, and 202 ± 13 g, respectively. These phenotypes were not different from the WF/NHsd phenotype (*P*-values = 0.4788, 0.9560, and >0.999, respectively).

We measured body mass of male and female *Mcs3^J^* and WF rats at 4, 8, and 12 weeks of age to determine the effect of *Mcs3* at different stages of male and female development (Fig. 4b) (Supplementary Table 3). Respectively, these ages coincided with prepuberty, puberty, and adult stages of development. For these experiments, male and female rats were housed in separate cages, but within the same environment (standard housing room) and not exposed to DMBA. There was no significant difference between strains for either biological sex at 4 weeks of age. Interestingly, *Mcs3^J^* females housed in rooms containing males and not exposed to DMBA had body mass phenotypes at 4, 8, and 12 weeks of age that were not different from WF females. However, at 8 weeks of age, males homozygous for *Mcs3^J^* alleles had a body mass of 149 ± 21 g (*n* = 30), which was significantly less than the WF male body mass of 186 ± 15 g (*n* = 31) (*P* < 0.0001). A significant difference between *Mcs3^J^* and WF male body mass was also observed at 12 weeks of age (244 ± 37 and 267 ± 29 g, respectively, *P* < 0.0001).

Females in rat mammary carcinogenesis studies were housed in environments that did not contain males. We questioned whether the effect of *Mcs3* on body mass seen at 23 weeks of age in DMBA-exposed females was due to the absence of males (Fig. 4a). To test this, we raised females in the absence males postweaning, but did not administer DMBA (Fig. 4c). In the absence of males, homozygous *Mcs3^J^* females (*n* = 24) had significantly lower body mass compared to WF/NHsd females (*n* = 15) at 8 weeks of age (114 ± 9 and 134 ± 5 g, respectively, *P* < 0.0001) and at 12 weeks of age (152 ± 13 and 178 ± 7 g, respectively, *P* < 0.0001) (Fig. 4c). Body mass statistics are contained in Supplementary Table 3.

**Fig. 5.**
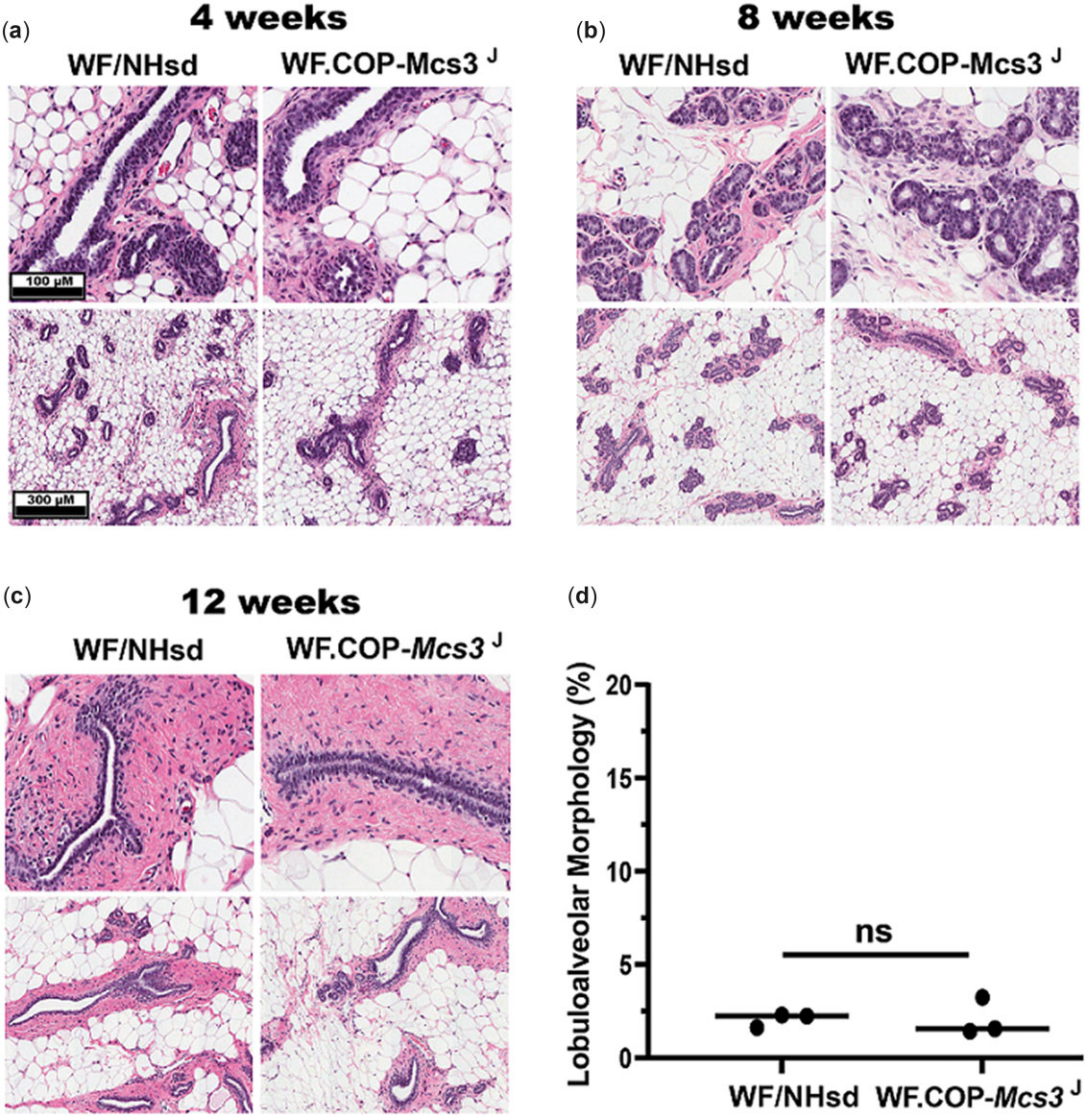
Mammary gland development of WF/NHsd and WF.COP-*Mcs3^J^* females raised in the presence of males appears normal. a–c) Representative H&E staining of virgin WF/NHsd (left) and WF.COP-*Mcs3^J^* (right) mammary glands at 4, 8, and 12 weeks of age. Images were captured at 40× magnification on an Aperio ImageScope CS2. a, b) Histology at 4 and 8 weeks is mixed population of TEBs and ductal structures embedded throughout an adipocyte matrix in both strains. c) Histology at 12 weeks is majority of mature ducts, with loss of TEBs, and minimal lobuloalveolar structures, which is representative of adult rat virgin mammary glands. d) Quantitative analysis of % lobuloalveolar morphology in mammary gland cross-sections at 12 weeks of age revealed no difference in lobuloalveolar area between WF/NHsd and WF.COP-*Mcs3^J^* mammary glands [2-tailed unpaired *t*-test *P* > 0.05 (ns)].

**Fig. 6.**
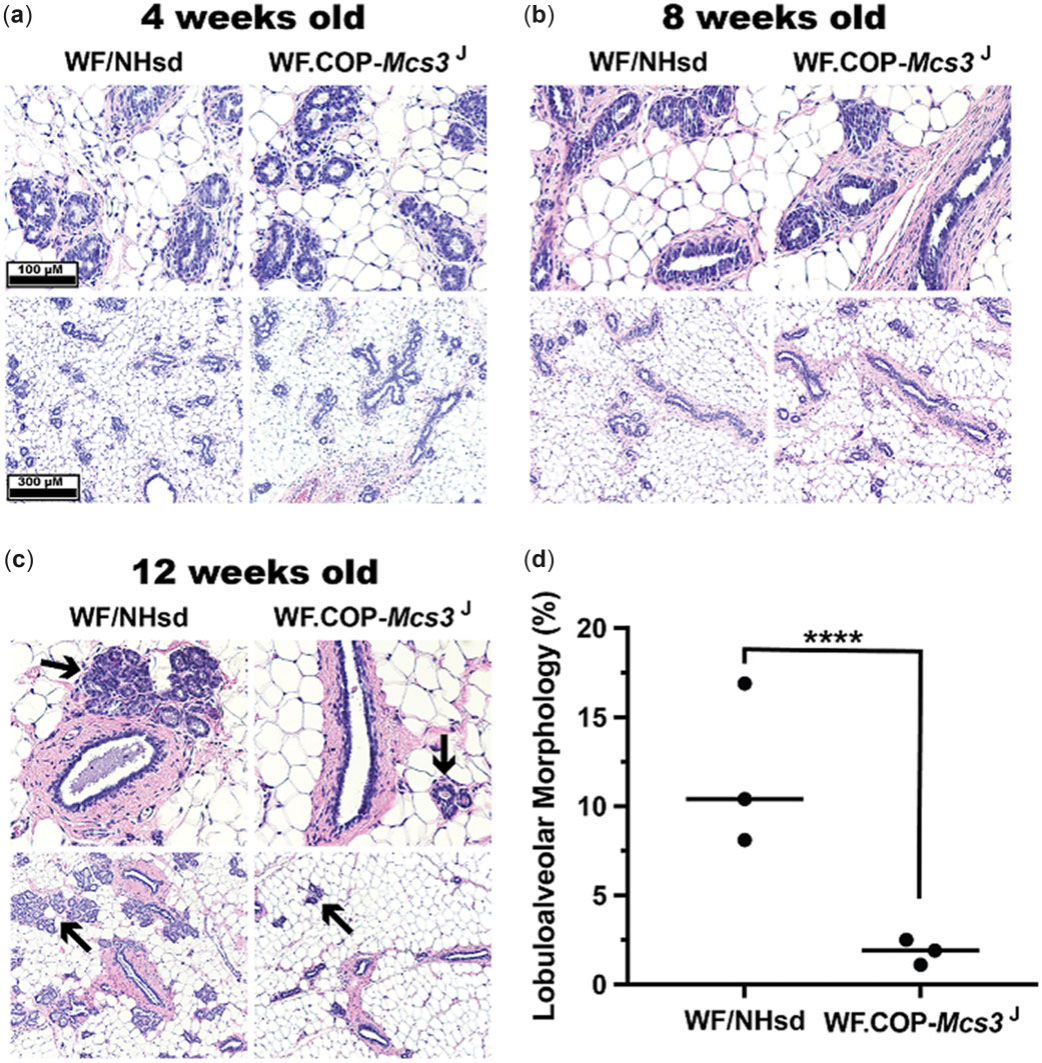
Effect of *Mcs3* on mammary gland development of females raised in the absence of males. Mammary carcinoma susceptible WF/NHsd, but not resistant WF.COP-Mcs3^J^ females had enhanced lobuloalveolar mammary gland histology at 12 weeks of age when raised in the absence of males. a–c) Representative H&E staining of WF/NHsd (left) and WF.COP-Mcs3^J^ (right) mammary glands at 4, 8, and 12 weeks of age. Images were saved at 40× magnification using an Aperio ImageScope CS2. a) Mammary gland histology at 4 weeks is mixed TEBs and ductal structures embedded throughout an adipocyte matrix in both strains. b) Histology at 8 weeks is mixed TEB structures and maturing ductal structures with more pronounced stroma than at 4 weeks of age in both strains. c) Histological analysis at 12 weeks of age reveals Mcs3^J^ females have mature ducts and minimal lobuloalveolar structures (black arrows), which are representative of an adult virgin mammary gland. On the other hand, age-matched WF females display greatly enhanced lobuloalveolar structures. d) Quantitative analysis of % lobuloalveolar morphology in mammary glands at 12 weeks of age revealed increased lobuloalveolar area in susceptible WF compared to Mcs3^J^ females (*n* = 3; 2-tailed unpaired *t*-test *P* < 0.0001).

Histological analysis was completed to determine if the *Mcs3^J^* allele influenced mammary gland development or morphology. Abdominal-inguinal mammary glands at 4, 8, and 12 weeks of age from females raised in the presence or absence of males were analyzed. Females from both *Mcs3^J^* and WF/NHsd strains in both housing conditions had similar terminal end bud (TEB) and ductal structure morphologies at 4 and 8 weeks of age with both having more pronounced stroma at 8 weeks (Figs. 5a/b and 6a/b). At 12 weeks of age, WF/NHsd and *Mcs3^J^* females raised in the presence of males exhibited loss of TEB structures, largely ductal histology, and little lobuloalveolar epithelium as typical of the adult virgin mammary gland (Fig. 5c). There was no quantitative difference in lobuloalveolar morphological area between strains (Fig. 5d). Adult *Mcs3^J^* and WF/NHsd females raised in the absences of males had dramatically different mammary gland histology at 12 weeks of age (Fig. 6c). The mammary cancer-susceptible WF strain had a more enhanced lobuloalveolar morphology, which was not observed in *Mcs3^J^* females. Quantification of H&E-stained mammary gland cross-sections revealed that the WF strain had 6-times more lobuloalveolar morphological area compared to *Mcs3^J^* females at 12 weeks of age (*P* < 0.0001, Fig. 6d).

### Comparative genomics of rat *Mcs3*

Rat *Mcs3* (7.2 Mb, *rs8149408* to *rs107402736*) was found to contain sequence orthologous to segments of human chromosomes *11* and *15* (Table 2). Rat *Mcs3* also contained genic sequence that mapped to gene orthologs on human chromosome *10* (Table 3). Rat and human reference genomes were searched to identify annotated genes in rat Mcs3 and human orthologous regions. There were 23 genes in common between rats and humans, 12 human-only, and 20 rat-only genes. A majority of the rat-only genes encode olfactory receptors not conserved in the human genome. Eleven of the 23 genes in common had published associations with breast cancer (Table 3) (Kovacs 2001; Martinez *et al.* 2008; Evensen *et al.* 2013; Milke *et al.* 2013; de Groot *et al.* 2014; Tervasmäki *et al.* 2014; Pangeni *et al.* 2015; Sheng *et al.* 2016; Kasoha *et al.* 2017; Kalra *et al.* 2018; Tolkach *et al.* 2018; Chen *et al.* 2019; Golmohammadzadeh *et al.* 2019; Kønig *et al.* 2019; Yu *et al.* 2019).

**Table 2.** Human genomic segments orthologous to rat *Mcs3*.^a^

Human chromosome position (GRCh38/hg38)	Rat *Mcs3* coverage (% of bases)	Rat *Mcs3* coverage (% of span)
chr15:80005820–82285404	12	31.6
chr15:83134545–84130720	4.4	13.2
chr11:89365341–89617253	1.1	4.2

a *Mcs3* position chr1:143700228–150929594 (assembly RGSC 6.0/Rn6).

**Table 3.** Breast cancer-associated genes located in human genomic segments orthologous to rat *Mcs3*.

Gene ID	Gene name	Locus	RefSeq gene function	References
*IL16*	*Interleukin 16*	*15q25.1*	Pleiotropic cytokine that functions as a chemoattractant and modulator of T-cell activation	Kovacs (2001) and Milke *et al.* (2013)
*FOLH1 (PSMA)*	*Folate hydrolase*	*11p11.12*	A glutamate carboxypeptidase	Kasoha *et al.* (2017) and Tolkach *et al.* (2018)
*BCL2A1* ^a^	*BCL2 related protein A1*	*15q25.1*	Reduces release of pro-apoptotic cytochrome c and blocks caspase activation. Direct transcription target of NF-kappa B	Kønig *et al.* (2019) and Yu *et al.* (2019)
*CYP2C19/Cyp2c6v1*	*Cytochrome P450 family 2 subfamily C member 19*	*10q23.33*	Monooxygenase involved in xenobiotic and estrogen metabolism	Kalra *et al.* (2018) and Tervasmäki *et al.* (2014)
*EFL1*	*Elongation factor like GTPase 1*	*15q25.2*	Enables GTPase activity and ribosome binding activity	Sheng *et al.* (2016)
*CYP2C8/Cyp2c79*	*Cytochrome P450 family 2 subfamily C member 8*	*10q23.33*	Monooxygenase involved in xenobiotic and estrogen metabolism	Golmohammadzadeh *et al.* (2019)
*ARNT2*	*Aryl hydrocarbon receptor nuclear translocator 2*	*15q25.1*	Binds DNA regulatory sequences in genes responsive to developmental and environmental stimuli	Martinez *et al.* (2008)
*CEMIP*	*Cell migration inducing hyaluronidase 1*	*15q25.1*	Positive regulation of protein phosphorylation and transport	Evensen *et al.* (2013)
*ABHD17C*	*Abhydrolase domain containing 17C*	*15q25.1*	Involved in protein depalmitoylation	Chen *et al.* (2019)
*TM6SF1*	*Transmembrane 6 superfamily member 1*	*15q25.2*	A sterol isomerase	de Groot *et al.* (2014)
*BNC1*	*Basonuclin 1*	*15q25.2*	Zinc finger protein present in basal cell layer of epidermis and germ cells. Regulatory role in keratinocyte proliferation and differentiation, and oocyte maturation	Pangeni *et al.* (2015)

a Rat *Bcl2a1* is not located at *Mcs3*, but at *RNO8:96551424-96559527* of rat reference genome *rn6.*

**Fig. 7.**
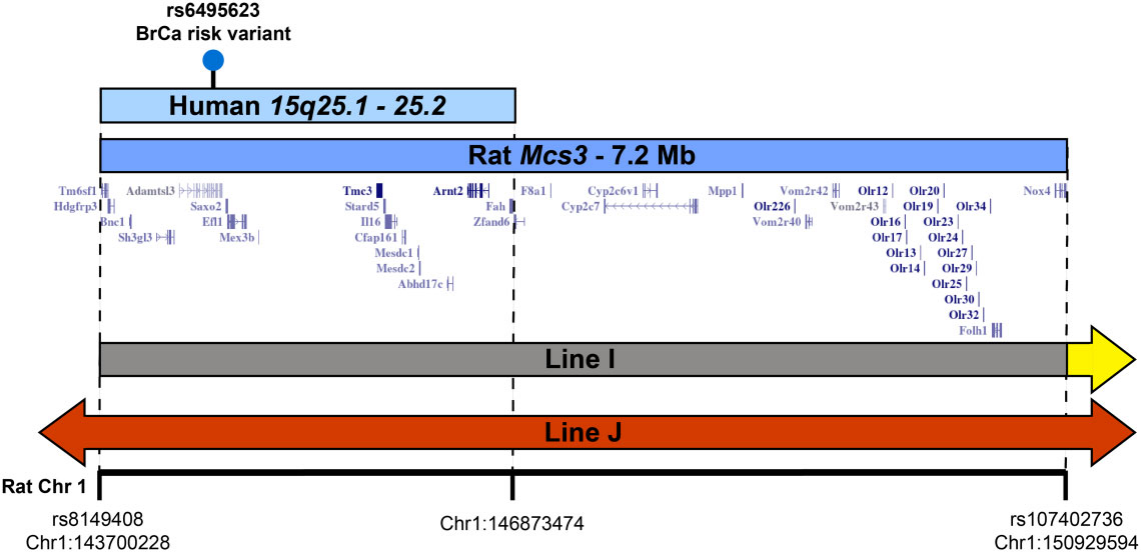
Transcript map of rat *Mcs3* and orthologous overlap with human *15q25.1-2*, a potential breast cancer risk locus. Map of *Mcs3* locus transcripts annotated by the UCSC rat genome browser (build version RGSC 6.0/rn6). The horizontal axis represents *RNO1* from bp 143,700,228–150,929,594, to which *Mcs3* was delimited. Mammary carcinoma-resistant WF.COP-Mcs3^J^ (red bar) and susceptible WF.COP-I (yellow arrow with a gray bar extension representing a segment of undetermined WF/COP genotype) are shown for reference. Vertical dashed lines demark the terminal ends of *Mcs3* (dark blue bar labeled “Rat *Mcs3* - 7.2 Mb”) and the respective rat genomic region that contains *Mcs3* overlap with orthologous human *15q25.1-25.2* (light blue bar labeled “Human *15q25.1 - 25.2”*). The lollipop within *15q25.1-25.2* marks the approximate location of associated breast cancer risk variant *rs6495623*.

**Fig. 8.**
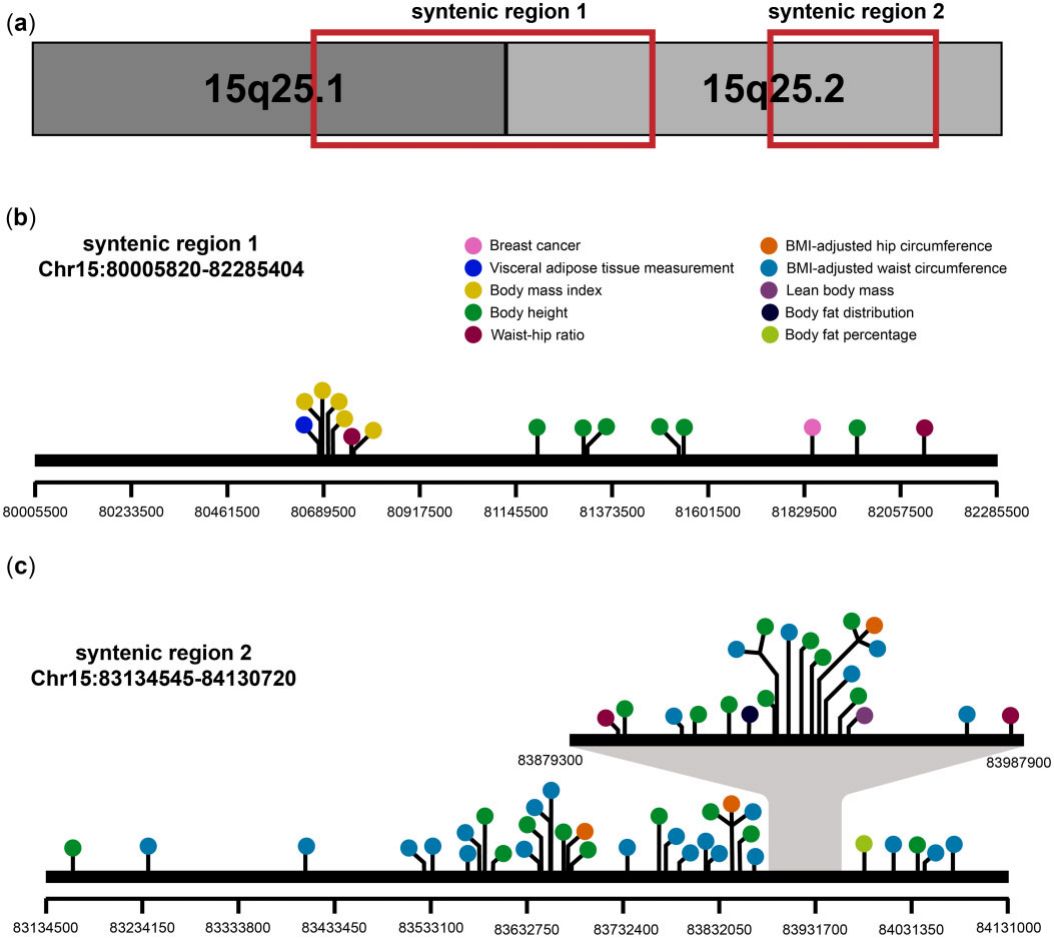
Lollipop maps of *Mcs3* orthologous human *15q25.1-25.2* and GWAS-nominated variants associated with body mass and other breast cancer-correlated traits. a) Relative location of human *15q25.1-25.2* syntenic regions orthologous to rat *Mcs3* (red boxes). b) Orthologous syntenic region 1 contains portions of *15q25.1* and *15q25.2* (chr15:80005820–82285404). c) Orthologous syntenic region 2 contains part of *15q25.2* (chr15:83134545–84130720). Black horizontal bars represent the genomic track with intervals indicating genomic position beneath. Each lollipop color represents a different trait as indicated in the legend inset. Variants with multiple trait associations share a stem.

A map of rat *Mcs3* transcribed genes and orthology with human *15q25.1-25.2* is depicted in Fig. 7. Human genome segments orthologous to rat *Mcs3* were queried for genetic associations to breast disease and cancer risk correlated traits using the NHGRI-EBI catalog of human genome-wide association studies. Human *15q25.2* variant *rs6495623* (hg38 chr15:81848308) was nominally associated to breast cancer risk with a p-value for association of 0.000871 (Hunter *et al.* 2007). Two of the human syntenic regions at *15q25.1-25.2* (chr15:80005820–82285404 and chr15:83134545–84130720) possessed 69 relevant body mass-associated variants (*P*-value for association <10^−7^) that were reported in genetic association studies of body mass index, visceral adipose tissue measurement, waist–hip ratio, BMI-adjusted hip circumference, BMI-adjusted waist circumference, lean body mass, body fat distribution, and body fat percentage (Fig. 8 and Supplementary Table 4).

## Discussion

Rat *Mcs3* was a previously mapped and physically confirmed mammary carcinoma resistance allele that, in this study, was further delimited from a 29.4- to 7.2-Mb segment of *RNO1* with orthology to 3 regions of the human genome. One of these segments, human *15q25.2*, contains a nominally associated breast cancer risk variant (*rs6495623*, Hunter *et al.* 2007). A variant located in *15q25.1*, *rs149479659*, is associated with ER+ breast cancer mortality (Escala-Garcia *et al.* 2019). Eight out of 11 genes located within rat *Mcs3* and human *15q25.1-25.2* orthologous sequence were found to have previously published functional associations to breast cancer (Table 3). Interestingly, human *15q25.1* variation has been associated with lung cancer susceptibility (Amos *et al.* 2008; Hung *et al.* 2008; Gu *et al.* 2012). The possibility of a genetic correlation between breast and lung cancer (*r_g_* = 0.18; *P* = 1.5 × 10^−6^) (Jiang *et al.* 2019; Rashkin *et al.* 2020) suggests that our *Mcs3* rat model could be used to investigate genetic mechanisms of breast and possibly lung cancer.

Rat *Mcs3* did not influence mammary tumor histopathology, as both *Mcs3* carcinoma resistance-associated and susceptible females developed papillary and cribriform mammary carcinomas. A majority of DMBA-induced rat mammary carcinomas are histologically defined as invasive papillary carcinomas and invasive cribriform carcinomas, comprising 32% and 27% of tumor histology, respectively (Russo 2015). A papillary histology is defined by branching fibrovascular cores throughout the lesion that contain variable degrees of stromal fibrosis and also support growth of papillary projections (Pal *et al.* 2010; Wei 2016; Rakha and Ellis 2018). A cribriform histology is defined by invasion of desmoplastic stroma by neoplastic epithelial cells that form nestlike tumor structures with a sieve-like appearance (Kadota *et al.* 2014; Russo 2015; Branca *et al.* 2017). Others have shown that DMBA-induced mammary tumors in pubescent, nulliparous rats, are adenocarcinomas of mammary ductal origin that develop primarily at TEBs and terminal ducts (Russo 2015). These intraductal and invasive ductal carcinomas are similar in histopathology to carcinomas observed in a majority of human breast malignancies (Russo 2015).

Mammary cancer-susceptible WF females had significantly higher lobuloalveolar morphology at 12 weeks compared to age-matched resistant WF.COP-*Mcs3^J^* females, but only when raised in the absences of males. This effect of the *Mcs3* COP allele introgressed into a WF genome is supported by similar findings from a study of 17β-estradiol exposure that reported the inbred COP strain had reduced lobuloalveolar mammary epithelia compared to the mammary cancer-susceptible ACI strain (Harvell *et al.* 2000). Whether the complete inbred COP decreased lobuloalveolar development phenotype seen by Shull and colleagues is controlled by the *Mcs3* locus remains to be determined. Our study mimicked effects of a continuous exposure to exogenous estradiol by housing females in socioenvironmental conditions without males present, which would be expected to cause hormone disruption. Although a causal relationship cannot be concluded from our study, it does suggest evidence to support the idea that a cancer-susceptible mammary gland may be innately more responsive to endogenous environmental components, such as hormonal perturbations, than a resistant mammary gland.

In addition to *Mcs3* associated mammary cancer resistance and lobuloalveolar development, *Mcs3* influenced body mass. Mammary cancer-resistant *Mcs3* females had lower body mass than susceptible WF females. Body mass and genetically correlated traits, such as obesity, are associated with breast cancer risk (Kwan *et al.* 2012; Chan *et al.* 2014; Greenlee *et al.* 2017). Specifically, obesity is associated with larger tumor size, positive lymph node status, shorter time to disease recurrence, and mortality (Loi *et al.* 2005; Rosenberg *et al.* 2009; Chan *et al.* 2014; Copson *et al.* 2015). A positive correlation between obesity and breast cancer risk in postmenopausal women has been reported (Li *et al.* 2006; Neuhouser *et al.* 2015; Sebastiani *et al.* 2016; Picon-Ruiz *et al.* 2017). This relationship is hypothesized to be due to adipose tissue being a main site of estrogen synthesis in postmenopausal women (Brouckaert *et al.* 2018) and higher concentrations of estrogen that would be caused by excess fat reserves (Ooi *et al.* 2019). These greater estrogen levels are thought to drive estrogen-dependent tumors, which is supported by the fact that most breast cancer cases in postmenopausal obese women are ER positive (Enger *et al.* 2000; Rosenberg *et al.* 2006; Suzuki *et al.* 2006; Ahn *et al.* 2007; Canchola *et al.* 2012). Inversely, there is a negative relationship between obesity and breast cancer risk in premenopausal women (Michels *et al.* 2006; Berstad *et al.* 2010; Harris, Willett, *et al.* 2011; Ooi *et al.* 2019). It has been speculated this might be due to less estrogen in circulation during menstrual cycling (Picon-Ruiz *et al.* 2017). It has been further suggested that this effect is reversed or lost upon adjusting for breast density (Boyd *et al.* 2006; Harris, Tamimi, *et al.* 2011; Engmann *et al.* 2019). Extra adipose tissue results in an inflammatory transcriptome and leads to the production of inflammatory cytokines, which creates a microenvironment conducive to cancer initiation, invasion, and metastasis (Dirat *et al.* 2011; Gilbert and Slingerland 2013; Picon-Ruiz *et al.* 2016). Conversely, a lower body mass, such as that observed in *Mcs3* females, could be protective toward disease development. This would positively correlate with what has been found in human epidemiological studies of body mass and female breast cancer risk. Future studies of body mass differences between mammary cancer-susceptible and -resistant *Mcs3* females could reveal body composition differences and corresponding physiological responses that contribute to mammary carcinoma resistance.

Many of the human GWAS-identified variants associated with body mass that are located within *15q25.1-25.2* reside in the *SH3 Domain Containing GRB2 Like 3*, *Endophilin A3* (*SH3GL3*), and *ADAMTS-like protein 3* (*ADAMTSL3*) genic region. This specific human gene region has not been shown to contain breast cancer risk-associated variants; however, the human body mass-associated variants in this region are relevant because mammary cancer-resistant *Mcs3* females had lower body mass than cancer-susceptible females. This suggests that if *Mcs3* controls both body mass and mammary cancer susceptibility, then these orthologous human regions may be doing the same.

The reduced body mass and mammary gland lobuloalveolar development phenotypes of *Mcs3* females compared to cancer-susceptible females was dependent on social environment, as *Mcs3* females had a lower body mass and lobuloalveolar phenotypes only when housed in an environment without males present. The only social environmental difference was the presence or absence of male pheromones, as males were never housed within the same cage with experimental females after weaning. Male pheromones are known to induce physiological and hormonal changes in the female endocrine and reproductive system (Rekwot *et al.* 2001). For example, male urinary pheromones have been demonstrated to increase plasma-luteinizing hormone and progesterone levels in females (Tsai *et al.* 1994; Tomioka *et al.* 2005). The endocrine system has a role in body weight regulation. Disruptions of this system could theoretically influence overall body mass. Furthermore, regulatory action of ERα has a known downstream effect on body mass (Musatov *et al.* 2007; Shi and Clegg 2009). Increased food intake and body mass have been demonstrated in ovariectomized rats, with restoration of normal eating behavior and body mass upon estradiol replacement (Asarian and Geary 2002; Shi and Clegg 2009). Furthermore, targeted deletion of ERα subunit has been reported to increase body mass and adiposity in mice (Heine *et al.* 2000). Estrogen modulates leptin synthesis and secretion, which regulates energy homeostasis and body mass (Machinal *et al.* 1999; Ainslie *et al.* 2001; Clegg *et al.* 2006). It remains to be determined if the *Mcs3*-mediated body mass difference, in the absence of males, is due to the disruption of endocrine signaling.

An effect of *Mcs3* on female body mass, dependent on the absence of males, is a classic example of a genotype–environment interaction (G × E, Fletcher and Dudbridge 2014). Strain-dependent differences among grouped females have been reported (Bruce 1963). Our results could translate to human GWAS designs by indicating that if allelic effect sizes are masked by environmental effects (Dempfle *et al.* 2008). For example, a significant association of *rs10483028* on chromosome *21q22.12* and breast cancer risk was detected in women with a body mass index below 25 kg/m^2^, and no association with disease was detected in women with a BMI of 30 kg/m^2^ or higher (Schoeps *et al.* 2014). There is also suggestive evidence of a G × E between the *Mcs3*-orthologous human *15q25.1* region and smoking behavior for lung cancer risk. Numerous lung cancer risk risk-associated variants in this region also associate with smoking behavior and intensity. It stands to reason that the disease risk alleles marked by these variants were discovered because of genotypes influencing smoking behavior (VanderWeele *et al.* 2012; Yu *et al.* 2012). Thus, future studies of breast cancer risk warrant ultrafine mapping of rat *Mcs3* and human *15q25.1-25.2* loci to identify causal disease variants and genes. These studies may need to consider a potential for G × E interactions in study designs.

There is a cluster of olfactory receptor genes at the distal end of rat *Mcs3*. It is plausible that 1 or more of these genes could be involved in *Mcs3* associated phenotype differences because it has been shown that effects of mammalian pheromones are prevented by excision of female olfactory bulbs (Parkes and Bruce 1961; Liberles 2014). Pheromonally induced changes in hormone signaling are known to modulate reproductive phenotypes. These include the induction of estrus cycling in noncycling females due to the presence of a male, known as the Whitten effect; pregnancy blocking of newly mated females due to a presence of strange or alien males, known as the Bruce effect; and suppression or inhibition of estrus cycling in grouped females, known as the Lee–Boot effect (Van Der Lee and Boot 1955; Bruce 1959; Whitten 1959; Ryan and Schwartz 1977). The latter was determined to cause entrance into pseudopregnancy and not short periods of anestrus. In our study, the advanced lobuloalveolar mammary gland morphology of susceptible females was consistent with pseudopregnancy morphologies and environmental conditions known to result in pseudopregnancies (Ryan and Schwartz 1977; Hvid *et al.* 2012). Pseudopregnancy is classically associated with failed fertilization, wherein the mechanical stimulus of copulation without fertilization results in a 10- to 12-day pseudopregnancy period characterized by a progestational state (Adler *et al.* 1970; Terkel 1986). However, during each estrus cycle, there is a brief period of sensitivity where ovarian corpus lutea can be activated and maintained (Terkel 1986). Socioenvironmental grouping of females after weaning without males is an initial corpus lutea-activating stimulus that allows a self-sustaining pseudopregnancy via elevated progesterone and positive feedback on luteotropic prolactin secretion (De Greef and Zeilmaker 1979; Terkel 1986).

In conclusion, rat *Mcs3* was delimited to a 7.2-Mb locus that controls mammary carcinoma susceptibility, body mass, and mammary gland morphology. Concordance between orthologous human and rat loci for these traits provides strong evidence that human genetic and rat mechanistic studies are warranted. Additional studies, including further mapping of *Mcs3* and functional characterization of positional candidates, will be needed to determine if *Mcs3* pleiotropy is explained by a single locus or a cluster of independent subloci. The *Mcs3* rat model developed in this study will be a valuable resource to identify causal genes and mechanisms of mammary cancer resistant that might be applicable to female breast cancer prevention. It will also be interesting to characterize the genotype by socioenvironmental interactions identified in this study, as findings may be applicable to human conditions including obesity, metabolic disorders, and mammary gland development windows of breast cancer susceptibility. Most importantly, these results provide strong rationale for further genetic analysis of the human orthologous locus for causal breast cancer susceptibility genes and variants.

## Supplementary Material

jkac288_Supplementary_Data

## Data Availability

All data are presented within this article and supplementary files. Supplemental material is available at G3 online.
