# Supplementary material for: Rat Mammary carcinoma susceptibility 3 (Mcs3) pleiotropy, socioenvironmental interaction, and comparative genomics with orthologous human 15q25.1-25.2
Source: G3 (Bethesda). 2022 Oct 31;13(1):jkac288. doi: 10.1093/g3journal/jkac288 (PMC9836357; doi:10.1093/g3journal/jkac288)
Supplement: jkac288_Supplementary_Data [file jkac288_supplementary_data.zip › Suppl/Table_S4_G3-2022-403740.docx]

**Table S4. Breast Cancer Risk Correlated Traits with Human GWAS Significant Variants in *15q25.1-25.2* Syntenic Segments Orthologous to Rat *Mcs3***

| Variant dbSNP ID | P-Value* | Trait | Genomic position | Gene/locus | Accession number |
| --- | --- | --- | --- | --- | --- |
| rs7165759 | 1 x 10^-10^ | visceral adipose tissue measurement | Chr15:80696259 | ABHD17C | NM_021214.2 |
| rs12914623 | 2 x 10-16 | body mass index | Chr15:80701229 | ABHD17C | NM_021214.2 |
| rs57783949 | 4 x 10-8 | body mass index | Chr15:80710515 | ABHD17C | NM_021214.2 |
| rs34769775 | 9 x 10-14 | body mass index | Chr15:80696831 | ABHD17C | NM_021214.2 |
| rs2759315 | 5 x 10-11 | body mass index | Chr15:80717305 | ABHD17C | NM_021214.2 |
| rs12593036 | 2 x 10-15 | body mass index | Chr15:80766311 | ABHD17C | NM_021214.2 |
| rs12593088 | 5 x 10-12 | Waist-hip ratio | Chr15:80766299 | ABHD17C | NM_021214.2 |
| rs11325 | 5 x 10-9 | Body height | Chr15:81308999 | IL16 | NM_004513.6 |
| rs4448892 | 2 x 10-12 | Body height | Chr15:81196722 | IL16 | NM_004513.6 |
| rs17875533 | 1 x 10-18 | Body height | Chr15:81305884 | IL16 | NM_004513.6 |
| rs11857976 | 2 x 10-30 | Body height | Chr15:81531197 | AC060809 | AC060809.1 |
| rs28463783 | 1 x 10-10 | Body height | Chr15:81954237 | AC104041 | AC104041.1 |
| rs2654210 | 6 x 10-8 | Waist-hip ratio | Chr15:82113300 | LINC01583 | NR_120367.1 |
| rs35056343 | 2 x 10-13 | Body height | Chr15:81543120 | AC060809 | AC060809.1 |
| rs2554380 | 9 x 10-7 | Body height | Chr15:83647132 | ADAMTSL3/  SH3GL3 | NM_207517.3/  NM_003027.5 |
| rs10906982 | 2 x 10-8 | Body height | Chr15:83899406 | ADAMTSL3 | NM_207517.3 |
| rs11259933 | 1 x 10-19 | Body height | Chr15:83911404 | ADAMTSL3 | NM_207517.3 |
| rs7183263 | 4 x 10-7 | Body height | Chr15:83904289 | ADAMTSL3 | NM_207517.3 |
| rs1383484 | 9 x 10-7 | Body height | Chr15:83854003 | ADAMTSL3 | NM_207517.3 |
| rs11259936 | 2 x 10-35 | Body height | Chr15:83911830 | ADAMTSL3 | NM_207517.3 |
| rs2401171 | 4 x 10-21 | Body height | Chr15:83888924 | ADAMTSL3 | NM_207517.3 |
| rs2257011 | 1 x 10-47 | Body height | Chr15:83597393 | SH3GL3 | NM_003027.5 |
| rs7162542 | 8 x 10-55 | body height, BMI-adjusted waist circumference, BMI-adjusted hip circumference | Chr15:83845538 | ADAMTSL3 | NM_207517.3 |
| rs4842924 | 7 x 10-10 | Lean body mass | Chr15:83918855 | ADAMTSL3 | NM_207517.3 |
| rs2585061 | 5 x 10-13 | Body height | Chr15:83678999 | ADAMTSL3 | NM_207517.3 |
| rs8024628 | 5 x 10-19 | body height, BMI-adjusted waist circumference, BMI-adjusted hip circumference | Chr15:83917711 | ADAMTSL3 | NM_207517.3 |
| rs950169 | 2 x 10-7 | Body height | Chr15:84037709 | ADAMTSL3 | NM_207517.3 |
| rs2562781 | 2 x 10-9 | BMI-adjusted hip circumference | Chr15:83643816 | ADAMTSL3/  SH3GL3 | NM_207517.3/  NM_003027.5 |
| rs4842838 | 6 x 10-27 | BMI-adjusted waist circumference, body height | Chr15:83913372 | ADAMTSL3 | NM_207517.3 |
| rs8030379 | 9 x 10-27 | BMI-adjusted waist circumference | Chr15:83922158 | ADAMTSL3 | NM_207517.3 |
| rs2030839 | 1 x 10-15 | BMI-adjusted waist circumference | Chr15:83915207 | ADAMTSL3 | NM_207517.3 |
| rs111470917 | 7 x 10-37 | Body height | Chr15:83916787 | ADAMTSL3 | NM_207517.3 |
| rs8038454 | 4 x 10-11 | Body height | Chr15:83670684 | ADAMTSL3 | NM_207517.3 |
| rs11856122 | 2 x 10-31 | Body fat distribution | Chr15:83907596 | ADAMTSL3 | NM_207517.3 |
| rs8031704 | 1 x 10-15 | Body fat percentage | Chr15:83982433 | ADAMTSL3 | NM_207517.3 |
| rs62025778 | 1 x 10-20 | BMI-adjusted waist circumference | Chr15:83776366 | ADAMTSL3 | NM_207517.3 |
| rs75821416 | 2 x 10-13 | BMI-adjusted waist circumference | Chr15:83657008 | ADAMTSL3 | NM_207517.3 |
| rs75666428 | 8 x 10-11 | BMI-adjusted waist circumference | Chr15:84012650 | ADAMTSL3 | NM_207517.3 |
| rs55685171 | 6 x 10-10 | BMI-adjusted waist circumference | Chr15:83757828 | ADAMTSL3 | NM_207517.3 |
| rs7169595 | 9 x 10-9 | BMI-adjusted waist circumference | Chr15:83736822 | ADAMTSL3 | NM_207517.3 |
| rs140574883 | 1 x 10-13 | BMI-adjusted waist circumference | Chr15:84074328 | AC027807 | AC027807.1 |
| rs141724470 | 6 x 10-9 | BMI-adjusted waist circumference | Chr15:84044702 | ADAMTSL3 | NM_207517.3 |
| rs11259926 | 7 x 10-21 | BMI-adjusted waist circumference | Chr15:83897496 | ADAMTSL3 | NM_207517.3 |
| rs4842918 | 1 x 10-62 | BMI-adjusted waist circumference | Chr15:83868247 | ADAMTSL3 | NM_207517.3 |
| rs4583201 | 5 x 10-11 | BMI-adjusted waist circumference | Chr15:83820297 | ADAMTSL3 | NM_207517.3 |
| rs113817743 | 4 x 10-9 | BMI-adjusted waist circumference | Chr15:83817917 | ADAMTSL3 | NM_207517.3 |
| rs4842926 | 1 x 10-44 | BMI-adjusted waist circumference | Chr15:83939736 | ADAMTSL3 | NM_207517.3 |
| rs8035886 | 2 x 10-34 | BMI-adjusted waist circumference | Chr15:83790608 | ADAMTSL3 | NM_207517.3 |
| rs77378263 | 3 x 10-14 | BMI-adjusted waist circumference | Chr15:83571369 | SH3GL3 | NM_003027.5 |
| rs2730075 | 6 x 10-12 | BMI-adjusted waist circumference | Chr15:83645689 | ADAMTSL3/  SH3GL3 | NM_207517.3/  NM_003027.5 |
| rs75340848 | 2 x 10-10 | BMI-adjusted waist circumference | Chr15:83420223 | AC103876 | AC103876.1 |
| rs2220856 | 5 x 10-22 | BMI-adjusted waist circumference | Chr15:83526475 | SH3GL3 | NM_003027.5 |
| rs10152514 | 5 x 10-14 | BMI-adjusted waist circumference | Chr15:83583309 | SH3GL3 | NM_003027.5 |
| rs2732151 | 8 x 10-10 | BMI-adjusted waist circumference | Chr15:83534933 | SH3GL3 | NM_003027.5 |
| rs4843136 | 2 x 10-8 | BMI-adjusted waist circumference | Chr15:83403098 | AC103876 | AC103876.1 |
| rs4842921 | 8 x 10-11 | BMI-adjusted wait to hip ratio | Chr15:83887871 | ADAMTSL3 | NM_207517.3 |
| rs185392832 | 2 x 10-8 | Body height | Chr15:83162219 | HDGFL3 | NM_016073.4 |
| rs6602982 | 3 x 10-42 | Body height | Chr15:83588992 | SH3GL3 | NM_003027.5 |
| rs118136305 | 2 x 10-16 | Body height | Chr15:83769541 | ADAMTSL3 | NM_207517.3 |
| rs2135880 | 3 x 10-222 | Body height | Chr15:83920861 | ADAMTSL3 | NM_207517.3 |
| rs11630762 | 5 x 10-10 | Waist to hip ratio | Chr15:83946227 | ADAMTSL3 | NM_207517.3 |

*P-value for association > 10^-7^

** Homo sapiens assembly GRCh38/hg38
