# Supplementary material for: Rat Mammary carcinoma susceptibility 3 (Mcs3) pleiotropy, socioenvironmental interaction, and comparative genomics with orthologous human 15q25.1-25.2
Source: G3 (Bethesda). 2022 Oct 31;13(1):jkac288. doi: 10.1093/g3journal/jkac288 (PMC9836357; doi:10.1093/g3journal/jkac288)
Supplement: jkac288_Supplementary_Data [file jkac288_supplementary_data.zip › Suppl/Table_S1_G3-2022-403740.docx]

**Table S1.** **Microsatellite DNA Markers used to screen for recombination events at *Mcs3* in *RN01***

| Marker ID | Forward Sequence | Reverse Sequence | Amplicon Position* | Amplicon size WF/COP (bp) |
| --- | --- | --- | --- | --- |
| D1Rat382 | GGCCGAATGCTTTCAATAGA | GGCATACATGCTCAAACTGC | 137503081-137503201 | 123/139 |
| D1Rat173 | GATCCCTTGACAAGCATGGT | GATGGAGGCAGTTTTTCCAA | 152519382-152519547 | 158/174 |
| D1Mit3 | ACTTGGTGAAGAAGAGTCAGGG | GATTTACTGTGCCTGTGGTTTT | 156446196-156446783 | 129/121 |
| D1Rat274 | TTGGTACACACACGCACATG | CTGGACATAAGGACAACTGGAA | 161677683-161677836 | 175/157 |
| D1Mgh8 | CCTCTGGATTCTGCCAGAAG | TTTCAAATGTACAGGCTGAAACA | 163796316-163796432 | 143/111 |
| D1Rat277 | TCTGGTCTTTACATGTATGTGCA | TTCACATCAGTTTTGGCCAC | 171716519-171716714 | 194/210 |

WF = Wistar Furth, COP = Copenhagen

* *Rattus norvegicus* genome build version RGSC 6.0/rn6
