# Supplementary material for: Rat Mammary carcinoma susceptibility 3 (Mcs3) pleiotropy, socioenvironmental interaction, and comparative genomics with orthologous human 15q25.1-25.2
Source: G3 (Bethesda). 2022 Oct 31;13(1):jkac288. doi: 10.1093/g3journal/jkac288 (PMC9836357; doi:10.1093/g3journal/jkac288)
Supplement: jkac288_Supplementary_Data [file jkac288_supplementary_data.zip › Suppl/Table_S2_G3-2022-403740.docx]

**Table S2. Single nucleotide variants DNA Markers used to screen for recombination events at *Mcs3* in *RN01***

| **dbSNP ID** | **Ensembl ID** | **Forward Sequence** | **Reverse Sequence** | **Amplicon Size (bp)** | **Amplicon Position*** | **Variant Position*** | **Variant** | **COP** | **WF** |
| --- | --- | --- | --- | --- | --- | --- | --- | --- | --- |
| *rs8149408* | *ENSRNOSNP2784088* | AGTATCTGCCCGGTGG | GGCAAACCGTCCTGAAA | 155 | 143700164-143700318 | 143700228 | G/A | A/A | G/G |
| *rs107402736* | *ENSRNOSNP2784133* | TGCTTCGCCTTAACCTG | TGCATGTCAGAAGGGAGA | 330 | 150929449-150929778 | 150929594 | G/A | G/G | A/A |
| *rs105307119* | *ENSRNOSNP2784267* | TGACCTCCGTGCTACC | ACCTGGTGTCGCTTCA | 253 | 165593736-165593988 | 165593914 | T/A | A/A | T/T |
| *rs105131702* | *ENSRNOSNP2784324* | CTGTCACCCCAGCACT | TGATTTGTCCCGGGGA | 281 | 171517184-171517464 | 171517317 | T/C | T/T | C/C |

WF = Wistar Furth, COP = Copenhagen

* *Rattus norvegicus* genome build version RGSC 6.0/rn6
