# Supplementary material for: Rat Mammary carcinoma susceptibility 3 (Mcs3) pleiotropy, socioenvironmental interaction, and comparative genomics with orthologous human 15q25.1-25.2
Source: G3 (Bethesda). 2022 Oct 31;13(1):jkac288. doi: 10.1093/g3journal/jkac288 (PMC9836357; doi:10.1093/g3journal/jkac288)
Supplement: jkac288_Supplementary_Data [file jkac288_supplementary_data.zip › Suppl/Table_S3_G3-2022-403740.docx]

**Table S3. Body Mass Summary Data of WF/NHsd and WF.COP *Mcs3* Strain J Breeders**

| **Strain** | **Sex** | **Housing** | **Age (weeks)** | **Mean (SD) weight (g)** | **N** |
| --- | --- | --- | --- | --- | --- |
| WF/NHsd | Male | Regular | 4 | 67 (11) | 31 |
| WF/NHsd | Male | Regular | 8 | 186 (15) | 27 |
| WF/NHsd | Male | Regular | 12 | 267 (29) | 37 |
| WF.COP Strain J | Male | Regular | 4 | 52 (6) | 17 |
| WF.COP Strain J | Male | Regular | 8 | 149 (21) | 28 |
| WF.COP Strain J | Male | Regular | 12 | 244 (37) | 29 |
| WF/NHsd | Female | Regular | 4 | 65 (11) | 22 |
| WF/NHsd | Female | Regular | 8 | 130 (8) | 31 |
| WF/NHsd | Female | Regular | 12 | 175 (14) | 38 |
| WF.COP Strain J | Female | Regular | 4 | 51 (8) | 20 |
| WF.COP Strain J | Female | Regular | 8 | 122 (13) | 29 |
| WF.COP Strain J | Female | Regular | 12 | 168 (18) | 23 |
| WF/NHsd | Female | Female Only | 8 | 134 (5) | 15 |
| WF/NHsd | Female | Female Only | 12 | 178 (7) | 15 |
| WF.COP Strain J | Female | Female Only | 8 | 114 (9) | 24 |
| WF.COP Strain J | Female | Female Only | 12 | 152 (13) | 24 |
