# Supplementary figures and images for: Rat Mammary carcinoma susceptibility 3 (Mcs3) pleiotropy, socioenvironmental interaction, and comparative genomics with orthologous human 15q25.1-25.2
Source: G3 (Bethesda). 2022 Oct 31;13(1):jkac288. doi: 10.1093/g3journal/jkac288 (PMC9836357; doi:10.1093/g3journal/jkac288)

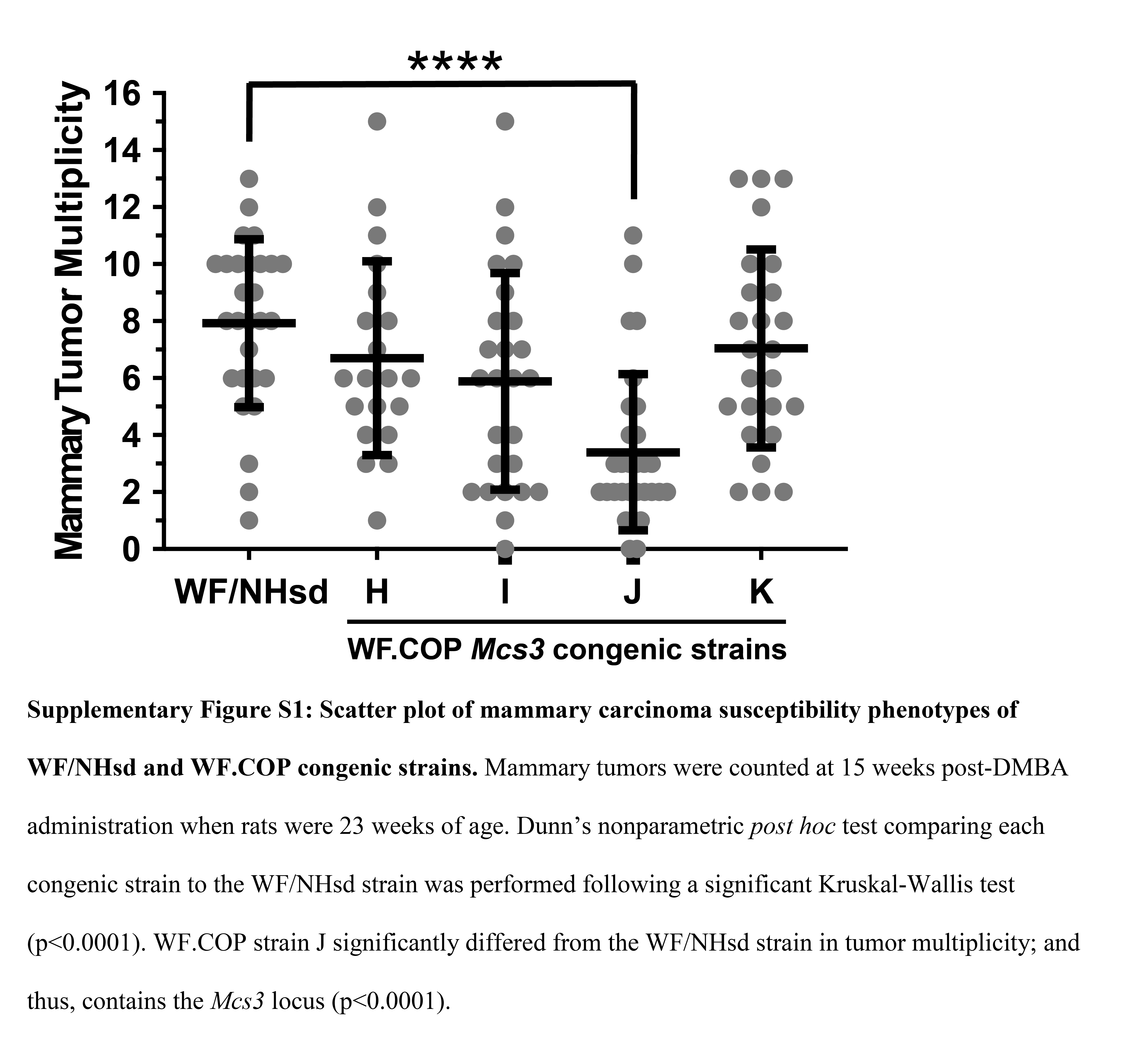

Supplement: jkac288_Supplementary_Data [file jkac288_supplementary_data.zip › Suppl/Figure_S1_G3-2022-403740.png]
